# Incremental residual polarization caused by aging in human skin

**DOI:** 10.1117/1.JBO.29.5.052912

**Published:** 2023-11-14

**Authors:** Viktor Dremin, Elena Zharkikh, Ivan Lopushenko, Zbignevs Marcinkevics, Alexander Bykov, Igor Meglinski

**Affiliations:** a University of Oulu, Opto-Electronics and Measurement Techniques Unit, Faculty of Information Technology and Electrical Engineering, Oulu, Finland; b Aston University, College of Engineering and Physical Sciences, Birmingham, United Kingdom; c University of Latvia, Department of Human and Animal Physiology, Riga, Latvia; d I.M. Sechenov First Moscow State Medical University, Human Anatomy and Histology Department, N.V. Sklifosovsky Institute of Clinical Medicine, Moscow, Russia

**Keywords:** polarization, hyperspectral imaging, human skin, aging, Monte Carlo simulation

## Abstract

**Significance:**

The study of the effect of aging on the optical properties of biological tissues, in particular polarization, is important in the development of new diagnostic approaches.

**Aim:**

This work aims to provide a comprehensive analysis of the factors and mechanisms that contribute to the alteration of skin polarization properties caused by aging, using polarization-sensitive hyperspectral imaging measurements and Monte Carlo simulation.

**Approach:**

Our investigation involved both experimental studies of *in vivo* human skin of volunteers of different ages and computational modeling that accounted for changes in the absorption and scattering properties of the skin model. Specifically, we analyzed alterations in the degree of linear polarization (DOLP) to better understand the impact of aging on skin polarization properties.

**Results:**

A statistically significant increase in the DOLP was found for the elderly group. At the same time, there was no correlation between changes in polarization and the calculated blood volume fraction parameter for different ages. According to the simulation results, it was also found that a change in the scattering properties of biological tissues has a more significant effect on the change in polarizing light compared to the change in absorption.

**Conclusions:**

The results of the work prove that the sensitivity of polarization imaging to age- or pathological-related skin changes may be primarily due to changes in scattering, which in turn is associated with changes in the collagen fibers of the dermis. The proposed technique shows promise for *in vivo* non-invasive real-time assessment of age-associated skin changes and can also be extended to monitor changes associated with the development of age-related pathologies.

## 1 Introduction

Recent research^1^^–^^7^ demonstrates the potential of bulk polarimetry as a standalone modality for the diagnosis of biotissues. Imaging with polarized light can provide rich information about the microstructure of samples as a result of the extremal sensitivity of the polarized light to their inner structure and composition.

In previous work,^8^ we showed a diagnostic approach with the ability to detect skin complications of diabetes mellitus (DM) in the earliest stages using a new polarization-sensitive hyperspectral imaging (HSI) system and machine-learning approaches. The developed system was shown to distinguish between the skin of DM patients and healthy volunteers, as well as between age-related and disease-related skin changes. One of the parameters capable of providing information about the changes in skin microstructure is the degree of linear polarization (DOLP).^1^ This parameter is calculated when probing radiation with the initial linear polarization and measuring two radiation intensities (co- and cross-polarized) reflected from the measuring object. The term co-polarized is used to define a linearly polarized backscattered component with the same orientation of polarization as an incident light; a cross-polarized component is polarized orthogonally with respect to the incident light polarization. It was experimentally demonstrated that DOLP parameter depends on the age and/or presence of diabetic complications assuming its dependence on tissue scattering changes.^8^ This work aims to provide a more detailed study of the causes and mechanisms of changes in the skin polarization properties caused by aging, using polarization-sensitive HSI measurements and Monte Carlo (MC) simulation.

**Fig. 1:**
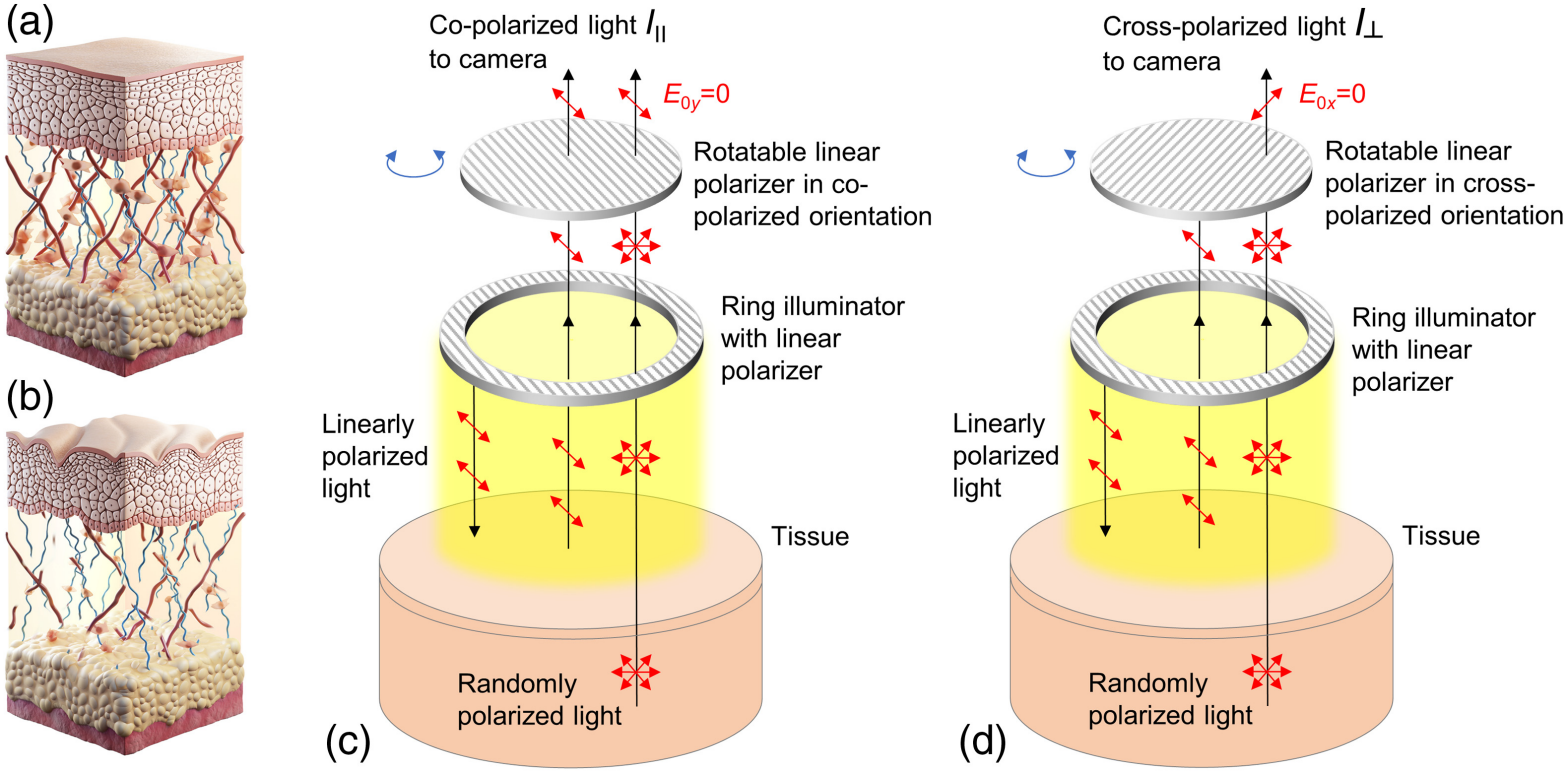
Schematic illustration of the age-related changes in human skin: (a) young and (b) aging skin. Broken collagen and elastin fibers and reduced fibroblast size are the hallmarks of structural changes in aging. Schematic representation of polarization imaging: (c) imaging of co-polarized light remitted from tissue and (d) imaging of cross-polarized light remitted from tissue eliminates signal from the superficial tissue layer.

The optical characteristics of skin as a biological tissue change significantly in the process of aging. As the skin ages, it undergoes structural and functional changes that are associated to a large extent with changes in collagen. Collagen is the main component of the extracellular matrix in mammals, including humans, so the structure and function of the skin depend primarily on its condition. As a person ages, the content of collagen in the dermis increases and its physical and chemical properties also change:^9^^–^^12^ the number and strength of inter- and intramolecular cross-links increases, whereas their elasticity and ability to swell decreases, collagen fibers develop resistance to collagenase, etc. [see Figs. 1(a) and 1(b)]. It is also known that the process of chemical modification of collagen that occurs with skin aging is greatly accelerated in certain medical conditions, such as DM.^13^^–^^15^

Age-related changes in the skin have a key influence on its optical properties, particularly changes in light scattering. Collagen fibers are one of the main scatterers in human skin tissue. Previous studies have shown the association between changes in skin scattering and age-related changes in skin. For example, Saadi et al.^16^ showed a positive correlation between reduced scattering coefficients and age in neonates. In contrast, Bosschaart et al., in their study, found no strong correlation between reduced scattering coefficients and age in neonates;^17^ the authors note, however, that they expect the parameter studied to increase at some point outside of the age range they examined, as published $\mu_{s}^{\prime }$ values are generally higher for adults.^18^ Jonasson et al.^19^ and Kono and Yamada^20^ reported a strong negative correlation between the age of the subjects and the reduced scattering coefficient. It is also known that collagen is one of the main skin constituents that define its birefringent properties.^21^ The magnitude of birefringence is related to the density and other mechanical properties of collagen fibers.^1^^,^^22^^,^^23^

Changes in the optical properties of turbid media, such as the values of the scattering coefficient and the absorption coefficient, strongly affect the polarization of the radiation interacting with the medium. The cut-off of a part of photons characterized by a large optical path due to their absorption leads to a significant increase in the DOLP for selective absorption bands caused by the presence of chromophores in the scattering medium.^24^ On the contrary, high scattering reduces the values of DOLP. Multiple scattering of radiation by turbid media affects the attenuation of the initial polarization of the propagating light at a rate that strongly depends on the optical properties of the scattering medium and the type of initial polarization (linear or circular).^25^

In this regard, age-related changes in the optical properties of biological tissues, particularly skin, will also affect polarization. In the following, we have experimentally and with the help of modeling traced the contribution of absorption and scattering to these changes.

## 2 Material and Methods

### 2.1 Polarization-Sensitive HSI of Human Skin

A specially designed compact imaging system based on a hyperspectral camera (Senop Optronics, Finland) with a microtunable Fabry–Pérot filter was used to carry out this study. Such a design of the system allows one to obtain a spectral resolution of about 6 to 10 nm in the total range of 510 to 900 nm.

A broadband illumination unit utilizing a 50 W halogen lamp is based on the fiber-optic ring illuminator (Edmund Optics, United States) that provides a uniform distribution of light intensity in the camera focal plane with the average irradiance of $4.3\pm 0.5\;\mathrm{mW}/{\mathrm{cm}}^{2}$ in the camera field of view on the skin surface. The use of the ring illuminator made it possible to combine the axes of illumination and detection. The illumination ring and the camera are equipped with rotatable broadband linear polarizers fixed at the crossed position for the reduction of specular reflection from the measured object. The three-dimensional (3D) model of the unit was implemented using computer-aided design software and printed with a 3D material printer. The considered measurement approach allows one to capture the reflected signal of the entire field of view at a certain waveband. The scanning is performed in the spectral domain. Generally, the constructed device is capable of recording spatially and spectrally resolved reflectance used for further artificial neural network (ANN) analysis. Thus, diffuse reflectance of the skin was recorded with the spectral step of 5 nm from the area of $8\times 8\;{\mathrm{cm}}^{2}$ at 1010×1010  pixels resolution of complementary metal-oxide semiconductor (CMOS) sensor. Normalized spatially resolved tissue reflectance was calculated as a pointwise ratio of dark-noise-corrected light intensity reflected from the skin and that reflected from the diffuse reflectance standard. A hypercube of the reflectance standard was recorded every time before the measurement of an object. Gray Spectralon (50% reflection, Labsphere, Inc., United States) was used as a calibration standard. The use of gray standard allows one to avoid the oversaturation of the CMOS sensor matrix possible for white reflectance standard (100% reflection) at the fixed integration time. A more detailed description of the system can be found in Refs. 8, 26, and 27.

The system utilized in this study was specially adapted for measurements of the degree of residual polarization of radiation reflected from biological tissue. For this purpose, the system was equipped with a rotating linear polarizer, which can be placed either parallel or perpendicular to the initial polarization of the light source. Figures 1(c) and 1(d) show a schematic representation of the measurement principle. Two hyperspectral images were acquired: $I_{∥}$ when the analyzer was parallel to the illumination and $I_{⊥}$ when the analyzer was perpendicular to the illumination. The hyperspectral cubes obtained were normalized to the images of the reflectance standard. The DOLP is defined as follows:

$$\mathrm{DOLP}=\frac{I_{∥}-I_{⊥}}{I_{∥}+I_{⊥}}.$$ (1)

For analysis, we proposed the polarization index (PI), which is the area under the curve of the DOLP spectrum, normalized by the number of spectral bands of the HSI system

$$\mathrm{PI}=\frac{1}{B}\overset{\lambda_{\max}}{\underset{\lambda_{i}=\lambda_{\min}}{\sum }}\mathrm{DOLP}(\lambda_{i}),$$ (2)

where $\lambda_{\min}=510\;\mathrm{nm}$ and $\lambda_{\max}=900\;\mathrm{nm}$ define the borders of the considered spectral range, and B=79 is the number of spectral bands of the HSI system. The normalization to the spectral bands number allows one to get the average value of DOLP that is ranged from 0 to 1 and unify measurements by various systems.

Experimental studies with conditionally healthy volunteers involving 32 people (9 males and 23 females, aged 22–76 years) were conducted. The experiments were carried out according to predetermined protocols approved at the local ethics committee meeting of April 3, 2019 (meeting minutes #28). All volunteers who participated in the experiment received a detailed description of the protocol and signed an informed consent form to participate. Hyperspectral images at the parallel and perpendicular orientation of the polarizers were recorded on the flat area of the proximal phalanx of the middle finger, which resulted in more than 100 recorded hyperspectral images. In earlier works, it was demonstrated that birefringent tissues randomize linearly polarized light.^28^ This is ensured by the fact that the dermal collagen fibers are somewhat randomly oriented with respect to the angle.^29^^,^^30^ If the tissue fibers are not near perfectly aligned, the orientation of linear polarization for different photons will rotate at different rates and the linearly polarized light will randomize. Thus, the influence of the position of the polarization axis will be minimized. At the same time, the orientation of the probing polarization in all of our measurements was aligned with the direction of the fingers of the hand.

To analyze the results obtained, volunteers who participated in the study were divided into two groups according to their age: the first group included 20 people aged 22 to 40 years, and the second group included 12 people over 40 years old.

The nonparametric Mann–Whitney U-test was used to confirm the reliability of the differences in the results. The values of p<0.01 were considered significant. The relationship of the parameters was estimated using Pearson’s correlation coefficient.

### 2.2 MC Simulations of Degree of Residual Polarization

Various MC models have been developed in the past to simulate the propagation of polarized light in scattering media.^31^^–^^33^ In general, in each MC approach a large amount ($N_{\mathrm{inc}}>10^{10}$) of MC-photons with pre-defined statistical weight ${\left(W_{0}\right)}_{j}$, $j=[1,\ldots ,N_{\mathrm{inc}}]$ is launched from the source and statistics is collected from those $N_{\mathrm{ph}}<N_{\mathrm{inc}}$ arrived on the detector. Within our model, the MC-photon scattering probability is defined by the medium scattering coefficient $\mu_{s}$ and direction at each scattering event is defined by the anisotropy parameter g via Henyey–Greenstein phase function^34^

$$p_{\mathrm{HG}}(\cos\;\theta^{\prime })=\frac{1}{4\pi }\frac{1-g^{2}}{{\left(1+g^{2}-2g\;\cos\;\theta^{\prime }\right)}^{3/2}},$$ (3)

where $\theta^{\prime }$ is the polar scattering angle in the local coordinate system of MC-photon. The absorption threshold for statistical weight is defined by Beer–Lambert–Bouguer law $W_{j}=(W_{0}{)}_{j}\;\exp(-\mu_{a}l_{j})$, where $W_{j}$ is detected MC-photon weight and $l_{j}$ corresponds to the j’th MC-photon path length within the turbid medium from the point of origin to the point of detection.^35^^,^^36^

To describe and track the polarization changes, recently developed MC approaches typically utilize Jones and/or Stokes–Mueller formalisms. In this paper, we use the Jones-based formalism to handle the linear polarization of light traveling through a random turbid medium.^37^^,^^38^ In particular, the Jones vector,

$$P=(\begin{array}{c} P_{x}\\ P_{y} \end{array}),$$

is assigned to each MC-photon at launch and is always orthogonal to its direction. For example, parallel |H⟩ and perpendicular |V⟩ polarization states of the MC-photon are described by vectors

$$|H\rangle =(\begin{array}{c} 1\\ 0 \end{array}),\;|V\rangle =(\begin{array}{c} 0\\ 1 \end{array}).$$

Evolution of the polarization vector P in a scattering medium is traced along the computed path of scalar MC-photon via the iterative solution of the Bethe–Salpeter equation reduced to the sequential transformations of the initial polarization^39^^–^^41^

PN=U^NU^N−1U^N−2,…,U^1P0, (4)

where $P_{0}$ is the initial polarization state of the MC-photon at launch, N is a number of scattering events for j’th MC-photon prior to detection, and $P_{N}$ is the resulting Jones polarization vector. We have also introduced a real 3×3 operator U^i, which employs MC-photon unit direction vector $s_{i}=[s_{iX},s_{iY},s_{iZ}]$ at i’th scattering event to trace polarization state^37^^,^^38^^,^^41^

U^i=(1−siX2−siX·siY−siX·siZ−siX·siY1−siY2−siY·siZ−siX·siZ−siX·siZ1−siZ2). (5)

This procedure ensures that each individual MC-photon remains fully polarized, but its polarization becomes a superposition of parallel and perpendicular states. By averaging over the MC-photon ensemble, the scattered light intensity components can be computed as^41^

$$I_{∥}=\frac{1}{N_{\mathrm{ph}}}\overset{N_{\mathrm{ph}}}{\underset{j=1}{\sum }}W_{j}P_{x_{j}}^{2}\Gamma_{R}^{N},$$ (6)

$$I_{⊥}=\frac{1}{N_{\mathrm{ph}}}\overset{N_{\mathrm{ph}}}{\underset{j=1}{\sum }}W_{j}P_{y_{j}}^{2}\Gamma_{R}^{N},$$ (7)

$$I_{+45{}^{\circ}}=\frac{1}{N_{\mathrm{ph}}}\overset{N_{\mathrm{ph}}}{\underset{j=1}{\sum }}\frac{W_{j}}{2}(P_{x_{j}}^{2}+P_{y_{j}}^{2}+2P_{x_{j}}P_{y_{j}})\Gamma_{R}^{N},$$ (8)

$$\begin{array}{c} I_{-45{}^{\circ}}=\frac{1}{N_{\mathrm{ph}}}\overset{N_{\mathrm{ph}}}{\underset{j=1}{\sum }}\frac{W_{j}}{2}(P_{x_{j}}^{2}+P_{y_{j}}^{2}-2P_{x_{j}}P_{y_{j}})\Gamma_{R}^{N}, \end{array}$$ (9)

where $\Gamma_{R}=2{\left(1+\overset{¯}{\cos^{2}\;\theta }\right)}^{-1}$ is the Rayleigh factor.^37^^,^^41^ Then DOLP can be defined as

$$\mathrm{DOLP}=\frac{\sqrt{{\left(I_{∥}-I_{⊥}\right)}^{2}+{\left(I_{+45{}^{\circ}}-I_{-45{}^{\circ}}\right)}^{2}}}{I},$$ (10)

where $I=I_{∥}+I_{⊥}=I_{+45{}^{\circ}}+I_{-45{}^{\circ}}$. In this work, initial |H⟩ polarization is employed and expression for DOLP can therefore be reduced to Eq. (1). Total internal reflection and/or refraction at the medium boundary are taken into account by splitting the MC-photon into the transmitted and reflected parts.^42^

A seven-layer skin model was used to calculate the DOLP spectra. The utilized model accounts for the in-depth distribution of blood in the dermis. The basics of the considered model have been previously described in Refs. 26, 38, 43, and 44. The absorption coefficient of i-layer takes into account the blood volume fraction (BVF) $C_{\text{blood}}$ in various vascular beds, oxygen saturation S, water content $C_{H_{2}O}$, and melanin fraction $C_{\mathrm{mel}}$^43^

$$\mu_{a}(\lambda )=\underset{i}{\sum }C_{i}\mu_{ai}(\lambda ).$$ (11)

The scattering coefficients of the layers are approximated on the basis of the combination of Mie and Rayleigh scattering.^43^^,^^45^ MC simulations were performed with noncoherent light, taking into account the real configuration of the HSI system. In this study, DOLP spectra were calculated on the basis of $N_{\mathrm{ph}}=10^{9}$ detected MC-photons.

Additionally, the previously developed GPU-accelerated MC approach^44^^,^^46^ was used for a routine simulation of detector depth sensitivity (also known as diagnostic volume or sampling volume) to check the penetration depth of the developed HSI setup. The skin model described above was also used for these calculations.

## 3 Results and Discussion

### 3.1 *In Vivo* Skin Imaging

**Fig. 2:**
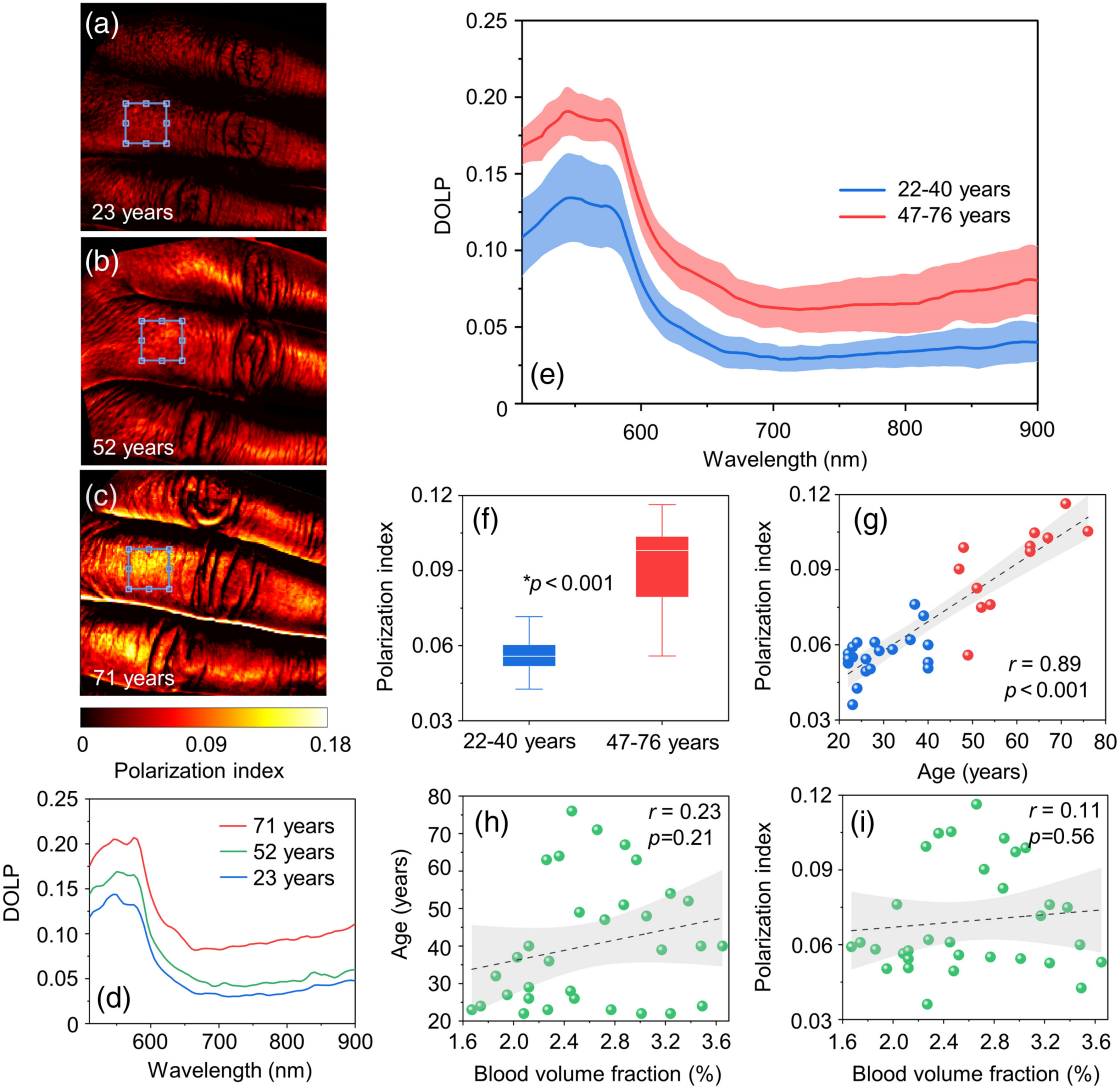
An example of the PI images (adapted from Ref. 8): (a) 23 years, (b) 52 years, and (c) 71 years. ROI indicates the averaging area to obtain the residual polarization spectra (d). (e) Residual polarization spectra for two age groups: up to 40 years and after 40 years, (f) comparison of PI among two age groups, (g) relationship between PI and age (blue circle: under 40 years; red circle: over 40 years), and (h) and (i) correlation between (h) age and BVF and between (i) PI and BVF. A Mann–Whitney U-test was used to identify differences between the two groups. The relationship of parameters was estimated using Pearson’s correlation coefficient.

Figures 2(a)–2(c) show examples of PI images and the corresponding DOLP spectra of the skin surface of the fingers for three female volunteers of different ages [Fig. 2(d)]. Here, the PI parameter was calculated using Eq. (2).

As mentioned above, for a detailed analysis, volunteers were divided into two age groups (22 to 40 and 47 to 76 years). Figure 2(e) shows the spectral distributions of the DOLP of radiation backscattered from skin at different ages. A general increase in the DOLP parameter is observed with age. A statistically significant increase of the PI parameter for elderly group (47 to 76 years) is observed [Fig. 2(f)]. In particular, the average value of PI for this group is 76% higher than for younger volunteers (22 to 40 years). Figure 2(g) shows the relationship between the PI and the age (r=0.89, p<0.001, Pearson correlation). In this case, linear regression best describes the relationship between DOLP and age. The shape of the spectrum up to 600 nm is primarily formed by peaks in the absorption of blood hemoglobin. Beyond the strong absorption bands, the curves in Fig. 2(e) show a decrease in the DOLP with increasing wavelength, which may be caused by less absorption of probing radiation by blood and other natural chromophores of the skin (in particular, melanin). Thus, in general, the spectral dependences of the DOLP are affected by the presence of different skin absorbers (blood, melanin, etc.) and scatterers (collagen, elastin, etc.).

Using the capabilities of our HSI system and ANN processing, we calculated the parameter of BVF^26^ and thus were able to analyze the dependence of DOLP on blood absorption. Previously, a strong positive correlation of these parameters was demonstrated when creating artificial erythema of the skin.^24^ In our studies, first, there was no correlation of this parameter with age [Fig. 2(h)]; second, there was no relationship with DOLP [Fig. 2(i)]. Therefore, it can be concluded that age-related changes in blood filling of the skin do not have a significant effect on DOLP, and it can be assumed that polarization is influenced by other changes in the optical properties during aging.

As for the other absorbers, in the visible spectral range considered, they are represented by melanin and yellow pigments (bilirubin and β-carotene). The ratio represented by Eq. (1) removes the melanin attenuation factor.^28^ Bilirubin and β-carotene are contained in low concentrations in the skin and can have high concentrations only in some diseases. At the same time, the main absorption is observed in the spectrum range up to 500 nm,^45^ which lies beyond the measurements of our system.

We assume that the differences shown are characterized by age-related collagen changes. The increase can be explained by the negative correlation between the skin scattering coefficient and the human age discussed in Sec. 1. For the polarized light, multiple scattering results in a loss of polarization. Thus, the low values of the scattering coefficient in older age lead to a lower depolarization and, as a consequence, to higher values of DOLP.

### 3.2 MC Simulation Results

**Fig. 3:**
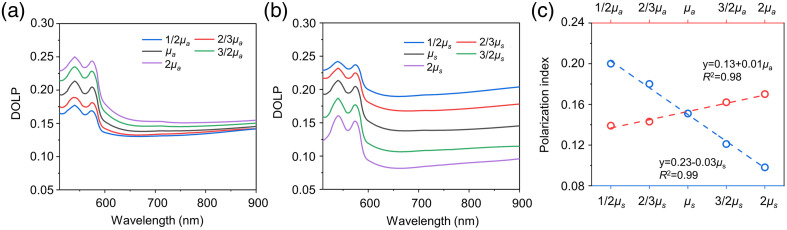
Simulated DOLP spectra for human skin at different values of dermis (a) absorption $\mu_{a}$ and (b) scattering $\mu_{s}$ coefficients. (c) Dependence of the PI on the dermis absorption (red circles) and scattering (blue circles) coefficients.

In addition, the contribution of absorption and scattering to changes in the DOLP spectra was estimated using MC modeling. The dependence of DOLP on the coefficients $\mu_{s}$ and $\mu_{a}$ coefficients was modeled (see Fig. 3).

In both cases, the default values of these coefficients were multiplied and divided by a factor of 1.5 and 2. With an increase in $\mu_{a}$, an increase in DOLP is observed [Fig. 3(a)]. Figure 3(b) shows that an increase in the value of $\mu_{s}$ in the absence of the influence of other factors leads to a corresponding decrease in the value of DOLP. The dependencies of the PI value on changes in $\mu_{s}$ and $\mu_{a}$ are shown in Fig. 3(c).

The simulation results demonstrate that a fourfold increase in scattering reduces DOLP by up to 50%. At the same time, with an identical change in absorption, DOLP increases only by 20%. Additionally, when the scattering changes, the DOLP spectrum changes over the entire spectral range, whereas absorption has a noticeable effect only in the region of strong hemoglobin absorption (500 to 600 nm). It can be seen from the experimentally recorded *in vivo* spectra [see Fig. 2(d)] that age-related changes in the spectrum are observed over the entire wavelength range. In this regard, it can be assumed that polarization is more affected by scattering. Age- and pathology-related changes in skin polarization^8^ can also be mainly explained by changes in this parameter.

**Fig. 4:**
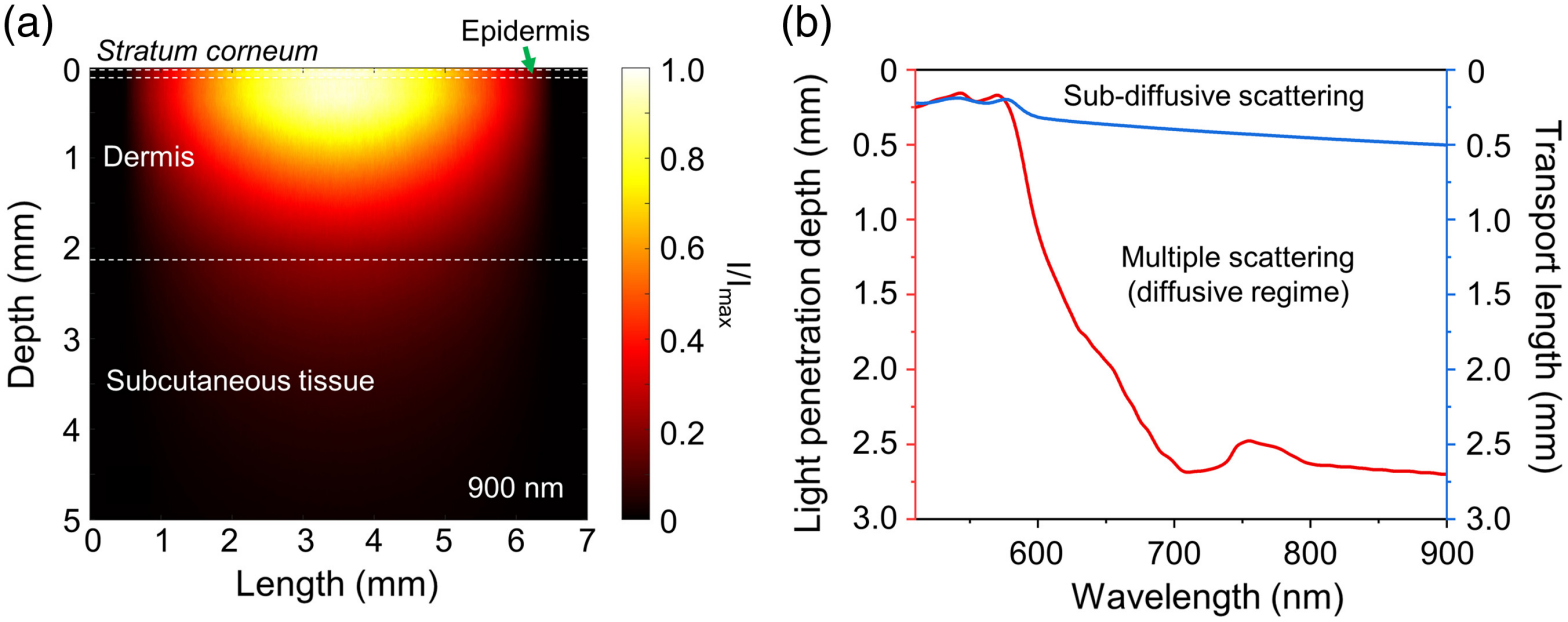
(a) The result of modeling the sampling volume for the experimental HSI setup for 900 nm wavelength. (b) Light penetration depth in the range from 510 to 900 nm at $1/e^{2}$ of the incident light intensity for the configuration of the used system and the estimated transport length.

At the same time, Ref. 47 shows that the greatest changes in the DOLP parameter occur with a small number of scattering events. With multiple scattering, the polarization changes are minimal. To check how sensitive our technology is to changes in polarization, we estimated the depth of radiation penetration into the skin using sampling volume modeling and calculated the transport mean free path^48^ using weighted averages of absorption and scattering coefficients. The results of the MC modeling of the sampling volume for the plane passing through the center of the entrance pupil of the HIS system and the wavelength of 900 nm are presented in Fig. 4(a). It is clearly seen that up to 600 nm the probing depth does not exceed 1 mm, whereas for the near-infrared (NIR) spectral range, it sharply increases and reaches up to 2.5 to 3 mm [Fig. 4(b)]. This confirms that the developed HSI system is primarily sensitive to the variations of blood content in the papillary dermis, upper blood net plexus, and is able to cover the top part of the reticular dermis. The transport length can be calculated approximately as $l^{*}=1/(\mu_{s}(1-g)+\mu_{a})$.

In Fig. 4(b), it can be seen that the transport length reaches 0.5 mm for the NIR range. The graph shows that when operating in the sub-diffusive regime in the spectral region up to 600 nm, the system will have the greatest sensitivity to changes in polarization. It is worth noting that most modern optical methods for probing biological tissues will be described by a similar scattering behavior.

Thus, the results of experimental studies and modeling show that the sensitivity of polarization spectroscopy or imaging to age- or pathological-related skin changes may be primarily due to a change in scattering, which in turn is associated with changes in the structure of collagen fibers of stromal tissues. The experimental system presented here and similar ones can be successfully used for the analysis of collagen-containing tissues in various fields of modern biomedicine, such as cancer and diabetes diagnostics, cosmetology, etc.

It is worth emphasizing that in this paper only changes in linear polarization were considered. Future research may be aimed at studying the changes in the full Stokes vector components. This will allow for a more detailed and comprehensive study of the relationship between changes in the absorption and scattering of biological tissues and their polarization properties. Indeed, a well-known feature is the dependence of the polarization decay rate on the type of initial polarization of the propagating light.^25^^,^^47^ In the Rayleigh scattering regime, the depolarization length for linearly polarized light exceeds that for circularly polarized light. In other words, linear polarization survives better in such scattering systems than circular polarization. In contrast, in the Mie scattering regime, the depolarization length for linearly polarized light is less than the similar parameter for circularly polarized light. Also, earlier in other studies, we demonstrated that the use of circular polarization can increase the dynamic range for various polarimetric parameters.^49^ Incorporating an additional measurement of circular polarization can enhance the sensitivity of the method for assessing early alterations in the polarization properties of the skin, potentially allowing for the quantitative differentiation between scattering and birefringence.^50^ Given that skin scattering is a complex interplay of Mie and Rayleigh scattering processes, delving into the behavior of different types of initial polarization holds considerable intrigue and potential for future biomedical applications. Understanding how various polarization states interact with skin tissue can provide valuable insights into the scattering mechanisms at play. This knowledge not only enriches comprehension of skin optics but also offers opportunities to develop more advanced and nuanced optical techniques for applications, such as non-invasive disease diagnostics, functional monitoring physiological changes, skin-care products, and drug delivery. Exploring the intricacies of polarization in skin scattering and tissue anisotropy (birefringence) paves the way to unlock new avenues for harnessing optical technology to further enhance healthcare and biomedical research.

## 4 Conclusion

The effect of scattering and absorption on the degree of residual polarization of backscattered radiation is studied in the case of probing of multiply scattering media by linearly polarized light. We studied DOLP changes in experimental studies of the skin of volunteers of different ages, as well as using modeling. *In vivo* studies have shown that apparently absorption associated with changes in blood filling does not affect the degree of residual polarization. We assume that the main influencing factor with increasing age is the scattering changes associated with changes in collagen structure. According to the simulation results, it was also found that a change in the scattering properties of biological tissues has a greater effect on the change in polarization compared to the change in absorption.

The results of the work prove that the polarization of light is sensitive to age and structural changes in the skin (collagen fibers of the dermis). Such changes may be an early sign of the development of different diseases and related complications. The proposed technique shows promise for *in vivo* non-invasive real-time assessment of age-related skin changes and can also be extended to monitor changes associated with the development of diabetes and other pathologies.

## Data Availability

Data underlying the results may be obtained from the authors upon reasonable request.
